# Does Overall Diet in Midlife Predict Future Aging Phenotypes? A Cohort Study

**DOI:** 10.1016/j.amjmed.2012.10.028

**Published:** 2013-05

**Authors:** Tasnime Akbaraly, Séverine Sabia, Gareth Hagger-Johnson, Adam G. Tabak, Martin J. Shipley, Markus Jokela, Eric J. Brunner, Mark Hamer, G. David Batty, Archana Singh-Manoux, Mika Kivimaki

**Affiliations:** a Department of Epidemiology and Public Health, University College London, London, UK; b Inserm, U1061 Montpellier, F-34093 France; c Université Montpellier I, Montpellier, F-34000, France; d Inserm U 1018, Assistance Publique-Hopitaux de Paris, France; e 1st Department of Medicine, Semmelweis, University Faculty of Medicine, Budapest, Hungary; f Institute of Behavioral Sciences, University of Helsinki, Finland; g Finnish Institute of Occupational Health, Helsinki, Finland; h MRC Centre for Cognitive Ageing and Cognitive Epidemiology, University of Edinburgh, UK

**Keywords:** Aging, Cognitive functioning, Dietary patterns, Diet quality indices, Mortality, Nutritional epidemiology, Overall diet, Physical functioning

## Abstract

**Background:**

The impact of diet on specific age-related diseases has been studied extensively, but few investigations have adopted a more holistic approach to determine the association of diet with overall health at older ages. We examined whether diet, assessed in midlife, using dietary patterns and adherence to the Alternative Healthy Eating Index (AHEI), is associated with aging phenotypes, identified after a mean 16-year follow-up.

**Methods:**

Data were drawn from the Whitehall II cohort study of 5350 adults (age 51.3 ± 5.3 years, 29.4% women). Diet was assessed at baseline (1991-1993). Mortality, chronic diseases, and functioning were ascertained from hospital data, register linkage, and screenings every 5 years and were used to create 5 outcomes at follow-up: ideal aging (free of chronic conditions and high performance in physical, mental, and cognitive functioning tests; 4%), nonfatal cardiovascular event (7.3%), cardiovascular death (2.8%), noncardiovascular death (12.7%), and normal aging (73.2%).

**Results:**

Low adherence to the AHEI was associated with an increased risk of cardiovascular and noncardiovascular death. In addition, participants with a “Western-type” diet (characterized by high intakes of fried and sweet food, processed food and red meat, refined grains, and high-fat dairy products) had lower odds of ideal aging (odds ratio for top vs bottom tertile: 0.58; 95% confidence interval, 0.36-0.94; *P* = .02), independently of other health behaviors.

**Conclusions:**

By considering healthy aging as a composite of cardiovascular, metabolic, musculoskeletal, respiratory, mental, and cognitive function, the present study offers a new perspective on the impact of diet on aging phenotypes.

In recent years, the impact of diet on age-related health outcomes has been investigated increasingly. Some studies have assessed diet using eating patterns derived through factor analyses without any “a priori” hypothesis.^1^ The findings have shown a protective impact of “Prudent,” “Mediterranean type,” and “Whole Food” diet against type 2 diabetes,^2^ cardiovascular diseases,^3,4^ and depressive symptoms.^5^ Other studies have used validated diet quality indices based on existing knowledge on “healthy eating,”^1^ such as the Mediterranean diet score.^6^ Adherence to this score has been associated with lower overall mortality, lower mortality from cancer and cardiovascular disease, and lower incidence of neurodegenerative diseases.^7,8^

The Alternative Healthy Eating Index (AHEI) is a validated index of diet quality, originally designed to provide dietary guidelines with the specific intention to combat major chronic conditions such as cardiovascular diseases.^9^ This index is a particularly relevant target for research on age-related morbidity. High scores on the AHEI have been shown to be associated with reduced risk of cardiovascular diseases^9^ and type 2 diabetes^10^ in a US population. Findings from the British Whitehall II study^11^ suggest that adherence to the AHEI also is related to an almost 2-fold higher odds of reversing the metabolic syndrome, a condition known to be a strong predictor of cardiovascular morbidity and mortality.^12^

In addition to examining diet as a modifiable determinant of age-related diseases, there is a need to consider aging as a process consisting of multiple health components. Identifying predictors of exceptional health in old age, for example, may provide new insights into optimal levels of established risk and protective factors. These might not necessarily be the same as those suggested in clinical guidelines for specific diseases (eg, coronary heart disease); research on ideal aging may inform new thresholds and targets for intervention. In terms of diet, a more holistic view on aging would complement the traditional approach and help identify dietary factors that not only prevent morbidity and mortality but also promote ideal aging (also referred to as “exceptionally healthy aging” or “successful aging”), which encompasses good functioning across different physical, mental, and cognitive domains.

In the present study, we therefore sought to assess the influence of diet measured in midlife by considering both adherence to healthy eating patterns and the AHEI recommendations, on a range of aging phenotypes assessed over a 16-year follow-up in a large British population of men and women.

## Methods

### Study Population

Participants in the Whitehall II study were London-based office staff, aged 35-55 years, who worked in 20 civil service departments at study inception.^13^ Baseline screening (phase 1: 1985-1988, n = 10,308) comprised a clinical examination and a self-administered questionnaire. Subsequent phases of data collection alternated between a clinical examination alongside a questionnaire survey and a postal questionnaire alone. Phase 3 (1991-1993, n = 8815) is considered the baseline for the purpose of this study because it represents the first assessment of dietary intakes. The target population of the present study comprised all participants at least 60 years of age by the end of follow-up (Phase 9: 2007-2009) and with no history of stroke, myocardial infarction, or cancer at Phase 3 (n = 7032). The University College London Ethics Committee approved the study. After the participants were given a complete description of the study, written informed consent was obtained from all participants.

As described in the flow chart diagram (Figure), the analytic sample consisted of 5350 participants aged 60 years or older at the final follow-up, with complete data on diet and main covariates at phase 3 and aging phenotype at phase 9.

### Dietary Assessment

At phase 3, participants were invited to fill a semi-quantitative food-frequency questionnaire containing 127 food items.^14,15^ The selected frequency category for each food item was converted to a daily intake. Nutrient intakes were computed by multiplication of the consumption frequency for each food by its nutrient content (for specified portions), and then the nutrient contributions from all foods were summed. Consumption frequency for multivitamin supplements also was collected. Nutrient values were calculated with the use of a computerized system developed for the Whitehall II dietary data. The validity and reliability of this version of the food-frequency questionnaire in terms of nutrient and food consumption have been documented in detail both in our cohort and in another independent UK cohort.^15,16^

### Outcome Ascertainment

Five aging outcomes were considered: 1) ideal aging, 2) nonfatal cardiovascular disease at follow-up, 3) cardiovascular death, and 4) noncardiovascular death. Those who did not belong to any of those categories were considered as the 5) natural (or normal) aging group. These health outcomes were ascertained from 3 follow-up screenings (Phase 5: 1997-1999; Phase 7: 2002-2004; and Phase 9: 2008-2009) plus records from national health registers.

#### Assessment of Nonfatal Cardiovascular Disease

Coronary heart disease status was based on clinically verified events and included myocardial infarction and definite angina. Nonfatal myocardial infarction was defined by using the World Health Organization Multinational Monitoring of Trends and Determinants in Cardiovascular Disease (MONICA) Project criteria^17^ and ascertained by using data from Whitehall II study resting electrocardiograms (ECGs) and hospital records of ECGs and cardiac enzyme levels obtained during acute myocardial infarction.^18^ Definite angina was identified by a questionnaire^19^ and corroborated by medical records or abnormal results on resting ECG, exercise ECG, or coronary angiography. Stroke was assessed using a self-reported measure of physician diagnosis.

#### Assessment of Cardiovascular Death and Noncardiovascular Death

Follow-up for mortality was performed through the national mortality register kept by the National Health Services Central Registry, using the National Health Services identification number assigned to each British citizen. The International Classification of Diseases, Ninth Revision (ICD-9), and 10th Revision (ICD-10) codes were used to define cardiovascular disease (ICD-9 390.0-458.9, ICD-10 I00-I99) mortality. Noncardiovascular disease mortality included all remaining deaths not classified as cardiovascular disease.

Criteria for ideal aging at age 60 years or over at the last follow-up (Phase 9) were:^20^

• Being alive.
• Absence of chronic diseases, such as coronary heart diseases, stroke, cancer (assessed using national cancer registry), and diabetes, which was determined by self-report of doctor diagnosis, use of antidiabetic medication, or oral glucose tolerance test (a fasting glucose ≥7.0 mmol/L, a 2-hour postload glucose ≥11.1 mmol/L).^21^
• Absence of mental health problems (defined by a score >42 in the mental health scale of the Short Form General Health Survey.^20,22^
• Good cardiometabolic, respiratory, musculoskeletal, and cognitive functioning: *cardiometabolic functioning* was assessed by: 1) age- and sex-standardized systolic blood pressure (average of 2 measurements in sitting position after 5 minutes of rest with the Omron HEM-907; Omron Healthcare Inc., Palatine, Ill) below the median at Phase 9, irrespective of antihypertensive medication use and 2) age- and sex-standardized fasting glucose; *respiratory functioning* consisted of above median age- and sex-standardized scores in forced expiratory volume in 1 second/height squared (FEV1/height^2^ in L/m^2^) at Phase 9;^23^ *musculoskeletal functioning* was assessed by above median age- and sex-standardized scores in walking speed over a clearly marked 8-foot walking course) at Phase 9;^24^ *good cognitive functioning* was defined as above median age- and sex-standardized cognitive score built from 5 cognitive tests at Phase 9^25^ chosen to provide a comprehensive assessment of cognitive function, which were: the Alice Heim 4-I, composed of 65 verbal and mathematical reasoning items of increasing difficulty;^26^ short-term verbal memory test (a 20-word free recall); 2 tests of verbal fluency (phonemic assessed via “S” words and semantic assessed via “animal” words);^27^ and the Mill Hill Vocabulary test, consisting of a list of 33 stimulus words ordered by increasing difficulty and 6 response choices.^28^ All functional measures were measured by a trained study nurse using standard protocols.

### Covariates

Smoking (non/former/current) and physical activity were assessed at Phase 3. Physical activity was assessed based on responses to questions on the frequency and duration of participation in mildly energetic (eg, weeding, general housework, bicycle repair), moderately energetic (eg, dancing, cycling, leisurely swimming), and vigorous physical activity (eg, running, hard swimming, playing squash). Participants were then classified as active (>2.5 h/week of moderate physical activity or >1 h/week of vigorous physical activity), inactive (<1 h/week of moderate physical activity and <1 h/week of vigorous physical activity), or moderately active (if not active or inactive).^29^

### Statistical Analysis

Dietary patterns were identified by performing principal component analysis of the 37 predefined food categories grouping the 127 food-frequency questionnaire items as detailed elsewhere.^5^ The factors were rotated by an orthogonal transformation (Varimax rotation function in SAS; SAS Institute, Cary, NC) to achieve a simple structure, allowing greater interpretability. Two dietary patterns were identified using multiple criteria: the diagram of Eigenvalues, the Scree plot, the interpretability of the factors, and the percentage of variance explained by the factors. The factor score for each pattern was calculated by summing intakes of all food groups weighted by their factor loadings. As detailed in Appendix Table 1 (online), the first pattern had high loading of items indicating intake of vegetables, fruits, and fish, labeled the “healthy-foods” dietary pattern. The second pattern, labeled “Western-type” diet, had high loadings of items indicating high consumption of fried food, processed and red meat, pies, sweetened desserts, chocolates, refined grains, high-fat dairy products, and condiments. Each participant received a factor score for both patterns. From the distribution of these factor scores, participants were categorized into tertiles for each pattern: the Tertile 1 group included participants with a factor score below the 33^rd^ percentile, the Tertile 2 group included participants with a factor score in the 33^th^-66^th^ percentile range and the Tertile 3 group included those with a factor score above the 66^th^ percentile.

The AHEI^9^ was scored on the basis of the intake levels of 9 components (Appendix Table 2, online): vegetables; fruits; nuts and soy; ratio of white meat (seafood and poultry) to red meat; total fiber; trans fat; ratio of polyunsaturated fat to saturated fat; long-term multivitamin use (≥5 vs < 5 years); and alcohol consumption. Each component had the potential to contribute 0-10 points to the total score, with the exception of multivitamin use, which contributed either 2.5 or 7.5 points (Appendix Table 2, online). All the component scores were summed to obtain a total AHEI score ranging from 2.5 to 87.5; higher scores corresponded to a healthier diet.

Characteristics of men and women according to the 4 aging outcomes and the normal aging category were compared using chi-squared test for categorical covariates and analysis of variance for quantitative covariates.

Logistic regression models were used to assess the association between dietary variables and each dichotomous aging outcome: ideal health, nonfatal cardiovascular disease (those who died from cardiovascular diseases were excluded from this analysis), cardiovascular death (the noncases included participants with no cardiovascular death over the follow-up but who may have had nonfatal cardiovascular diseases), and noncardiovascular death. Dietary variables consisted of dietary patterns (the “healthy-foods” and the “Western-type” diet) and the AHEI score, all of which were first categorized into tertiles (the lowest tertile used as the reference) to assess the association of the dietary variables with the 4 outcomes. These analyses were repeated using dietary variables as continuous standardized *z*-scores (mean = 0, standard deviation [SD] = 1), with odds ratios expressed per 1-SD increment in the dietary variable. Analyses were adjusted successively for age, sex, total energy intake (Model 1), and health behavior: smoking and physical activity (Model 2).

In a sensitivity analysis, we performed multinomial logistic regression to analyze associations between the 2 dietary patterns, AHEI score, and a 5-category outcome (ie, ideal aging, nonfatal cardiovascular disease, cardiovascular death, noncardiovascular death, normal aging) to test whether competing risk might have biased the findings obtained from separate logistic regression models; the natural aging category represented the noncase group.

All analyses were conducted using SAS software, version 9.

## Results

A total of 3775 (70.6%) men and 1575 (29.4%) women with baseline mean age 51.3 (SD 5.3) years were included. Compared with participants excluded (n = 1682), those included were less likely to be women (*P* <.0001), older (*P* = .002), and were more likely to have a higher AHEI score (*P* = .02); no significant difference was found for dietary patterns between these 2 groups. Of the 5350 participants at follow-up, 4% met the ideal aging, 12.7% developed a nonfatal cardiovascular disease, 2.8% died from cardiovascular disease, and 7.3% from noncardiovascular causes over the mean 16-year follow-up. The remaining 73.2% followed a natural aging course. Participant characteristics as a function of these aging phenotypes are presented in Table 1.

### Dietary Patterns

Results of the associations between “healthy-foods” and “Western-type” dietary patterns and aging outcomes are presented in Table 2. The “healthy-foods” diet (incorporating high intake of vegetables, fruits, and fish) was inversely associated with noncardiovascular mortality after adjusting for sex, age, total energy intake (Model 1, odds ratio [OR] per 1-SD increment 0.76; 95% confidence interval [CI], 0.68-0.84). However, this association was attenuated after further adjustment for health behaviors such as smoking status and physical activity (Model 2, OR 0.90; 95% CI, 0.79-1.01).

Participants in the top tertile of the “Western-type” diet (ie, a diet characterized by fried food, processed food and red meat, pies, sweetened desserts, chocolates, refined grains, high-fat dairy products, and condiments) had substantially lower odds for ideal aging compared with participants in the bottom tertile after adjustment for age, sex, and total energy intake (Model 1: OR 0.52; 95% CI, 0.33-0.82) and health behaviors (Model 2: OR 0.58; 95% CI, 0.36-0.93). A higher score of the “Western-type” diet also was associated with higher odds of both cardiovascular and noncardiovascular mortality (ORs per 1-SD increment 1.53; 95% CI, 1.16-2.01 and 1.36; 95% CI, 1.14-1.61, respectively) in the age, sex, and total energy intake-adjusted model, but those associations were attenuated after further adjustments.

To further examine the robustness of the association between “Western-type” diet and ideal aging, we performed subsidiary analyses for each component characterizing the ideal aging phenotype. Participants in the highest tertile of “Western-type” dietary pattern, compared with those in the bottom tertile, were more likely to have poorer musculoskeletal (OR for below-median walking speed = 1.45; 95% CI, 1.14-1.84) and cognitive functioning (OR for below-median test score = 1.58; 95% CI, 1.27-1.97). No significant association was found between “Western-type” diet and the indicators of cardiometabolic and respiratory functioning and mental health.

### The AHEI Score

Results of the association between the AHEI score and aging outcomes are presented in Table 3. After adjusting for age, sex, and total energy intake, high adherence to the AHEI recommendations was associated with decreased cardiovascular and noncardiovascular mortality, but not with nonfatal cardiovascular morbidity. The association between the AHEI score and ideal aging was nonlinear; compared with participants in the bottom AHEI tertile, those in the intermediate tertile had higher odds of ideal aging, while being in the highest tertile of AHEI and the continuous AHEI score was not significantly associated with ideal aging. Further adjustment for other health behavior did not modify those associations.

To examine potential competing risk bias, the association between dietary variables and aging phenotypes was examined using multinomial regression, allowing the estimation of odds of ideal aging and unhealthy aging outcomes within a single analytic setting, with natural aging as the common reference point for all 4 outcomes. Findings similar to that in the main analysis suggest that competing risk bias is unlikely (Appendix Table 3, online).

## Discussion

Complementing previous studies that have investigated the association between dietary behaviors and specific age-related diseases, the present report aimed to extend the findings by considering aging as a multi-component process. While our results indicate that low adherence to healthy recommendations of the AHEI guidelines is associated with increased premature death, the “Western-type” diet significantly reduced the likelihood of achieving ideal health at older ages, which incorporates cardiovascular, metabolic, musculoskeletal, respiratory, mental, and cognitive components. These associations were independent of other health behaviors such as physical activity and smoking.

We first assessed the association between “healthy-food” dietary pattern and aging phenotypes. This dietary pattern is characterized by intake of vegetables, fruits, and fish, and a beneficial effect on aging was expected due to the high content of antioxidants in fruits and vegetables (cf. the free radical theory of aging) and the high long-chain omega-3 polyunsaturated fatty acids content in oily fish, a major component of neuron membranes with vascular properties.^30^ However, in the present study, no significant association was observed between the “healthy-food” dietary pattern and the 4 aging outcomes. These results contrast with the literature showing protective effect of vegetarian diet or very low meat intake diet on human health and mortality*.*^31^ Of the 5350 participants, only 159 reported that they “never or rarely” consumed meat. Owing to low numbers of meat noneaters and the fact that we were not able to precisely define whether these participants were vegetarian or not, the interpretation of this null finding should be cautious; it is possible that the statistically nonsignificant associations observed between the “healthy-food” dietary pattern and subsequent aging phenotypes indicate an absence of beneficial impact of diet close to vegetarian diet, or alternatively is a consequence of lack of statistical power.

Our results showing that a diet matching a “Western-type” dietary pattern in middle age may be a risk factor for not achieving ideal healthy aging constitute a novel finding. The “Western-type” diet is composed of high consumption of fried food, processed and red meat, pies, sweetened desserts, chocolates, refined grains, high-fat dairy products, and condiments, and was to be associated particularly with the musculoskeletal and cognitive components of aging. In previous studies, “Western-type” (also labeled as “processed food”) diet has been associated with depressive symptoms^5^ and cognitive performance.^32^ The “Western-type” diet identified in our cohort is very close to the original “Western” pattern defined in the American population,^1^ which has been shown to be associated with higher risk of inflammation.^33^ At this stage, however, the mechanisms underlying the association between the “Western-type food” dietary pattern and lower odds of ideal aging remain unclear and need further investigation.

To complement our analysis of dietary patterns, which are obtained through statistical modeling of empirical data to describe the dietary behaviors of the studied population, we examined the impact of the AHEI dietary guidelines. Our findings confirm the links of the AHEI with reduced cardiovascular and noncardiovascular mortality reported previously in the present cohort.^14^ Our analyses did not suggest a significant association with nonfatal cardiovascular morbidity, while adherence to the AHEI has been suggested to reduce the risk of type 2 diabetes and cardiovascular disease in American cohorts.^9,10^ In terms of ideal aging, the role of AHEI remains unclear and requires further research.

Our data present some limitations. The main drawback concerns the generalizability of our findings. Whitehall II study participants are mainly white, office-based civil servants who are not fully representative of the British population.^13^ Moreover, for the present analysis, participants with missing values were excluded. Compared with the excluded, those included were less likely to be younger, to have a higher AHEI score, and better health outcomes. This may lead to an underestimation of the observed associations. The assessment of dietary intake using a semi-quantitative food-frequency questionnaire that only covered specific foods also constitutes a limitation. This method is recognized to be less precise than dietary assessment by the food diary method. However, we have shown previously in this study population that nutrient intake estimated by the food-frequency questionnaire method is correlated with biomarker levels and intake estimates from the generally more accurate 7-day diary.^15^ Finally, with observational data the possibility remains that unmeasured confounders may explain at least part of the observed association. Overall, the present study needs to be replicated in other cohorts to confirm the role of dietary patterns and adherence to AHEI guidelines in subsequent aging phenotypes.

Despite these limitations, the present study is unique by studying aging phenotypes based on validated clinic-based measures and medical records in a large cohort followed over a 16-year period. Furthermore, by analyzing ideal aging and unhealthy aging outcomes within a single analytic setting, we were able to avoid overlap of the different aging health components and reduce competing risk bias. We showed that specific dietary recommendations such as the one provided by the AHEI may be useful in reducing the risk of unhealthy aging, while avoidance of the “Western-type foods” actually might improve the possibility of achieving older ages free of chronic disease and remaining highly functional. A better understanding of the distinction between specific health behaviors that offer protection against diseases and those that move individuals towards ideal aging may facilitate improvements in public health prevention packages.

## Figures and Tables

**Appendix Table 1:** Factor Loading⁎ for High Loading Items (≥0.40)† on the Two Dietary Patterns Identified Using Principal Component Analysis at Baseline

	1^st^ Pattern: “Healthy-foods”	2^nd^ Pattern: “Western-type”
Leafy vegetables	0.69	—
Other vegetables	0.67	—
Tomatoes	0.59	—
Salad dressing	0.50	—
Fruits	0.49	—
Fish	0.46	—
Cruciferous vegetables	0.44	—
Fried food	—	0.56
Processed meats	—	0.52
Quiche/pie	—	0.47
Chocolate and sweets	—	0.46
Desserts/biscuits	—	0.45
Condiments	—	0.45
High fat dairy products	—	0.42
Refined grain	—	0.42
Red meat	—	0.41

Two dietary patterns were identified using multiple criteria: the diagram of Eigenvalues, the Screeplot, the interpretability of the factors, and the percentage of variance explained by the factors.

⁎ Factor loadings represent the correlation between the food groups and the dietary pattern.^12^

† Values <0.30 were not listed in order to simplify interpretation of the factors.

**Appendix Table 2:** Construction of AHEI Scores in 5350 Participants

Components	Criteria for Minimum Score	Criteria for Maximum Score	Possible Score Range	AHEI Scores in the Participants⁎
				M ± SD
Vegetable (serving/day)	0	5	0-10	5.6 (2.9)
Fruit (serving/day)	0	4	0-10	5.9 (3.1)
Nuts and soy (serving/day)	0	1	0-10	3.2 (3.0)
Ratio of white to red meat	0	4	0-10	5.1 (2.8)
Total fiber† (% of energy)	0	24	0-10	7.6 (3.0)
Trans fat (% of energy)	≥4	≤0.5	0-10	8.4 (2.7)
Ratio of PUFA to SFA	≤0.1	≥1	0-10	5.2 (2.7)
Duration of multivitamin use	<5 year	≥5 year	2.5-7.5	4.2 (2.4)
Alcohol serving/day, men	0 or >3.5	1.5-2.5	0-10	4.7 (3.7)‡
Alcohol serving/day, women	0 or >2.5	0.5-1.5	0-10	
Total score			2.5-87.5	50.0 (12.0)

AHEI = Alternative Healthy Eating Index; PUFA = polyunsaturated fatty acids; SAF = saturated fatty acids.

⁎ Each AHEI component contributed from 0 to 10 points to the total AHEI score, except the multivitamin component which was dichotomous and contributing either 2.5 points (for nonuse) or 7.5 points (for use) A score of 10 indicates that the recommendations were fully met, whereas a score of 0 represents the least healthy dietary behavior. Intermediate intakes were scored proportionately between 0 and 10.

† The original components of the index include cereal fiber, because cereal fiber was not available in our nutrient data set, we adapted the score by replacing it with total fiber.

‡ Mean score for men and women combined.

**Appendix Table 3:** Association between the Two Dietary Patterns, AHEI Scores, and the Four Aging Outcomes (n = 5350)

Dietary Pattern	Ideal Aging		Nonfatal CVD		Fatal CVD		Non-CVD Death		*P* Value
	OR	95% CI	OR	95% CI	OR	95% CI	OR	95% CI	
“Healthy-foods” pattern									
Model 1									
Tertile 1	1	reference	1	reference	1	reference	1	reference	
Tertile 2	1.23	0.87-1.74	0.97	0.79-1.20	0.63	0.42-0.95	0.67	0.52-0.87	
Tertile 3	1.16	0.80-1.69	1.06	0.86-1.32	0.67	0.44-1.02	0.73	0.56-0.96	
Effect per 1 SD	1.07	0.93-1.24	1.03	0.95-1.13	0.85	0.70-1.03	0.83	0.73-0.94	.008
Model 2									
Tertile 1	1	reference	1	reference	1	reference	1	reference	
Tertile 2	1.23	0.87-1.74	0.97	0.79-1.20	0.63	0.42-0.95	0.67	0.52-0.87	
Tertile 3	1.08	0.74-1.58	1.15	0.92-1.43	0.78	0.51-1.21	0.86	0.66-1.14	
Effect per 1 SD	1.04	0.90-1.21	1.06	0.97-1.16	0.93	0.77-1.13	0.90	0.80-1.02	.19
“Western-type” pattern									
Model 1									
Tertile 1	1	reference	1	reference	1	reference	1	reference	
Tertile 2	0.92	0.65-1.30	0.85	0.68-1.06	1.36	0.87-2.12	0.88	0.66-1.17	
Tertile 3	0.54	0.34-0.85	1.08	0.82-1.41	1.68	0.96-2.95	1.23	0.88-1.74	
Effect per 1 SD	0.77	.061-0.95	1.09	0.95-1.25	1.58	1.20-2.08	1.38	1.16-1.64	<10-4
Model 2									
Tertile 1	1	reference	1	reference	1	reference	1	reference	
Tertile 2	0.94	0.66-1.33	0.83	0.67-1.04	1.25	0.80-1.95	0.81	0.61-1.08	
Tertile 3	0.58	0.36-0.94	1.02	0.78-1.34	1.40	0.80-2.47	0.96	0.68-1.35	
Effect per 1 SD	0.83	0.66-1.05	1.03	0.89-1.18	1.35	1.02-1.78	1.16	0.97-1.38	.05
AHEI score									
Model 1									
Tertile 1	1	reference	1	reference	1	reference	1	reference	
Tertile 2	1.52	1.07-2.16	0.85	0.69-1.04	0.48	0.32-0.72	0.67	0.52-0.86	
Tertile 3	1.16	0.80-1.68	0.98	0.80-1.21	0.49	0.32-0.74	0.60	0.46-0.78	
Effect per 1 SD	1.09	0.95-1.27	0.97	0.89-1.06	0.70	0.59-0.83	0.75	0.67-0.83	<10-4
Model 2									
Tertile 1	1	reference	1	reference	1	reference	1	reference	
Tertile 2	1.41	0.99-2.01	0.90	0.73-1.10	0.54	0.35-0.81	0.76	0.59-0.98	
Tertile 3	1.04	0.71-1.52	1.07	0.87-1.32	0.59	0.38-0.90	0.74	0.56-0.96	
Effect per 1 SD	1.04	0.90-1.21	1.01	0.93-1.11	0.77	0.65-0.92	0.83	0.74-0.92	.006

AHEI = Alternative Healthy Eating Index; CI = confidence interval; CVD = cardiovascular disease; OR = odds ratio. Natural Aging was the “noncase” category for all aging phenotype outcomes presented. *P* for heterogeneity. Multinomial logistic regression to analyze associations between dietary patterns, AHEI score, and the 5-category aging outcome: 1) ideal health, 2) nonfatal cardiovascular disease at follow-up, 3) cardiovascular death, 4) noncardiovascular death, and 5) natural (or normal) aging (the noncase category for each of the other categories). Model 1: Adjusted for age, sex and total energy intake. Model 2: As Model 1 + additionally adjusted for other heath behavior: smoking habits and physical activity.

**Figure:**
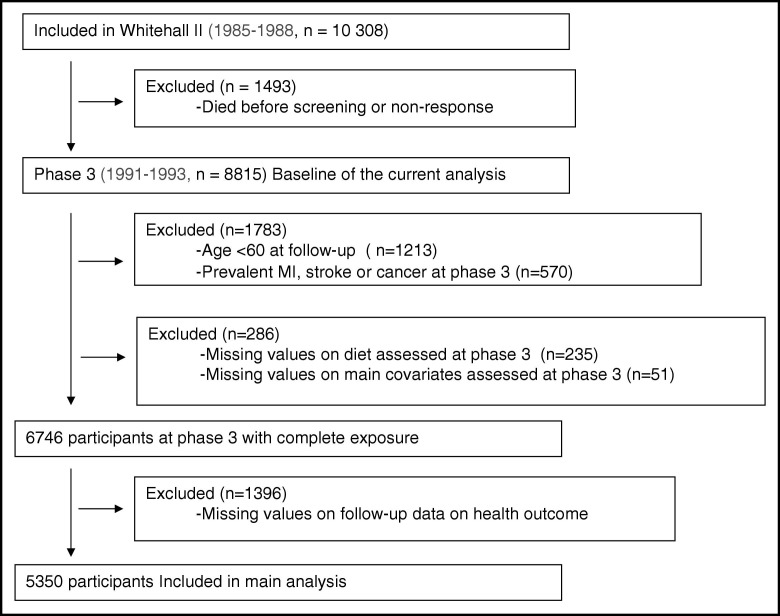
Flow chart of sample selection of the 5350 Whitehall II participants.

**Table 1:** Characteristics of Participants (n=5350) by Aging Phenotype, the Whitehall II study⁎

Characteristic	Ideal Aging	Nonfatal CVD	CVD Death	Non-CVD Death	Natural Aging†	*P* Value
	n = 213	n = 680	n = 149	n = 392	n = 3916	
Baseline						
Age, years	49.8 (5.1)	53.1 (5.2)	54.7 (4.9)	53.7 (5.3)	50.7 (5.1)	<.001
Sex, % women	31.5	24.4	24.8	32.9	30.0	.01
Total energy intake, kcal/day	2137 (618)	2148 (681)	2051 (638)	2099 (774)	2082 (605)	.08
Smoking habits, %						
Never	62.0	46.9	37.6	38.3	51.0	<.001
Former	34.7	36.0	36.2	32.9	36.3	
Current	3.3	17.1	26.2	28.8	12.7	
Physical activity, %						
Inactive	16.4	23.7	28.9	27.3	19.3	<.001
Moderately active	30.0	27.3	31.5	28.1	27.7	
Active	53.5	49.0	39.6	44.6	53.0	
AHEI score, %						
Tertile 1	25.8	33.4	49.0	41.1	32.4	<.001
Tertile 2	41.8	31.0	25.5	30.6	33.8	
Tertile 3	32.4	35.6	25.5	28.3	33.8	
“Healthy-foods” pattern, %						
Tertile 1	28.6	30.9	41.6	38.5	33.2	.04
Tertile 2	36.6	28.8	28.9	28.8	33.9	
Tertile 3	34.7	32.6	29.5	32.6	32.9	
“Western type” pattern, %						
Tertile 1	34.7	32.2	29.5	34.2	33.5	.04
Tertile 2	38.0	29.3	36.2	29.3	34.0	
Tertile 3	27.3	37.0	34.2	36.5	32.5	
Follow-up						
Coronary heart diseases, n (%) of cases	0 (0)	145 (25.1)	—	—	0 (0)	N/A
Stroke, n (%) of cases	0 (0)	175 (28.4)	—	—	0 (0)	N/A
SBP, mm Hg	112.9 (9.2)	125.3 (16.9)	—	—	127.7 (16.3)	N/A
Cancer, n (%) of cases	0 (0)	71 (10.5)	—	—	542 (13.9)	N/A
Type 2 diabetes, n (%) of cases	0 (0)	128 (18.8)	—	—	486 (12.4)	N/A
Lung function (FEV_1_/height^2^), L/m^2^	1.11 (0.18)	0.91 (0.23)	—	—	0.94(0.22)	N/A
Walking speed, m/s	1.41 (0.23)	1.11 (0.31)	—	—	1.15 (0.29)	N/A
Cognitive function, z-score	0.85 (0.6)	−0.22 (1.1)	—	—	−0.01 (0.97)	N/A
SF-36 mental health	56.1 (4.2)	53.4 (9.0)	—	—	53.8 (8.3)	N/A

AHEI = the Alternative Healthy Eating Index; CVD = cardiovascular disease; FEV = forced expiratory volume; SBP = systolic blood pressure; SF-36 = the Short Form 36 General Health Survey. From the distribution of these factor scores, participants were categorized into tertiles for each pattern: the Tertile 1 group included participants with a factor score below the 33^rd^ percentile, the Tertile 2 group included participants with a factor score in the 33^th^-66^th^ percentile range and the Tertile 3 group included those with a factor score above the 66^th^ percentile.

⁎ Numbers are means and SDs unless otherwise specified.

† Those alive at the end of follow-up without ideal health or nonfatal CVD.

**Table 2:** Association of the 2 Dietary Patterns at Baseline with 4 Aging Outcomes at Follow-up (n=5350), the Whitehall II Study

Dietary Pattern at Baseline	Aging Outcome at Follow-up											
	Ideal Aging			Nonfatal CVD			CVD Death			Non-CVD Death		
	OR	95% CI	*P* Value	OR	95% CI	*P* Value	OR	95% CI	*P* Value	OR	95% CI	*P*-Value
Healthy-foods diet												
Model 1												
Tertile 1	1	Ref		1	Ref		1	Ref		1	Ref	
Tertile 2	1.28	0.90-1.81	.17	1.00	0.81-1.23	.99	0.66	0.44-0.98	.04	0.70	0.54-0.90	.005
Tertile 3	1.19	0.82-1.73	.35	1.10	0.89-1.35	.39	0.66	0.43-1.01	.05	0.61	0.47-0.80	<.0001
Effect per 1 SD	1.08	0.94-1.25	.27	1.05	0.96-1.15	.26	0.86	0.71-1.04	.10	0.76	0.68-0.84	<.0001
Model 2												
Tertile 1	1	Ref		1	Ref		1	Ref		1	Ref	
Tertile 2	1.20	0.85-1.70	.30	1.05	0.85-1.30	.64	0.74	0.49-1.12	.15	0.77	0.59-1.00	.04
Tertile 3	1.08	0.75-1.57	.68	1.17	0.95-1.46	.14	0.76	0.51-1.20	.25	0.85	0.65-1.12	.25
Effect per 1 SD	1.04	0.90-1.21	.59	1.08	0.99-1.18	.09	0.93	0.77-1.13	.46	0.90	0.79-1.01	.07
Western-type diet												
Model 1												
Tertile 1	1	Ref		1	Ref		1	Ref		1	Ref	
Tertile 2	0.94	0.67-1.33	.73	0.87	0.70-1.08	.21	1.42	0.91-2.20	.12	0.90	0.68-1.18	.44
Tertile 3	0.52	0.33-0.82	.005	1.08	0.83-1.41	.56	1.66	0.95-2.89	.07	1.23	0.87-1.72	.24
Effect per 1 SD	0.73	0.58-0.91	.006	1.07	0.93-1.22	.33	1.53	1.16-2.01	.003	1.36	1.14-1.61	<.0001
Model 2												
Tertile 1	1	Ref		1	Ref		1	Ref		1	Ref	
Tertile 2	0.96	0.68-1.37	.84	0.85	0.68-1.06	.16	1.33	0.85-2.08	.21	0.83	0.63-1.10	.19
Tertile 3	0.58	0.36-0.93	.02	1.02	0.78-1.34	.86	1.35	0.77-2.37	.29	0.99	0.70-1.38	.94
Effect per 1 SD	0.81	0.64-1.03	.08	1.02	0.89-1.17	.79	1.33	1.00-1.76	.05	1.15	0.96-1.37	.12

CI = confidence interval; CVD = cardiovascular disease; OR = odds ratio. Model 1: Adjusted for age, sex and total energy intake. Model 2: Model 1 + additionally adjusted for smoking habits and physical activity. Results are from logistic regression models estimating the association of dietary patterns assessed at baseline with 4 aging outcomes at follow-up: ideal health (n = 213), nonfatal cardiovascular disease (n = 680), cardiovascular death (n = 149), and noncardiovascular death (n = 392). From the distribution of these factor scores, participants were categorized into tertiles for each pattern: the Tertile 1 group included participants with a factor score below the 33^rd^ percentile, the Tertile 2 group included participants with a factor score in the 33^th^-66^th^ percentile range and the Tertile 3 group included those with a factor score above the 66^th^ percentile.

**Table 3:** Association of Adherence to AHEI at Baseline with 4 Aging Outcomes at Follow-up (n = 5350), the Whitehall II Study

AHEI Score at Baseline	Aging Outcome at Follow-up											
	Ideal Aging			Nonfatal CVD			CVD Death			Non-CVD Death		
	OR	95% CI	*P* Value	OR	95% CI	*P* Value	OR	95% CI	*P* Value	OR	95% CI	*P* Value
Model 1												
Tertile 1	1	Ref		1	Ref		1	Ref		1	Ref	
Tertile 2	1.63	1.15-2.30	.006	0.87	0.71-1.07	.19	0.51	0.34-0.78	.001	0.68	0.53-0.88	.004
Tertile 3	1.23	0.85-1.78	.28	1.04	0.85-1.28	.69	0.51	0.34-0.77	.001	0.72	0.55-0.94	.01
Effect per 1 SD	1.13	0.98-1.31	.10	1.00	0.92-1.09	.99	0.72	0.61-0.86	<.0001	0.82	0.73-0.93	.002
Model 2												
Tertile 1	1	Ref		1	Ref		1	Ref		1	Ref	
Tertile 2	1.48	1.04-2.09	.02	0.92	0.74-1.13	.40	0.58	0.37-0.84	.006	0.79	0.61-1.02	.07
Tertile 3	1.07	0.73-1.55	.73	1.12	0.91-1.38	.28	0.60	0.39-0.92	.02	0.75	0.57-0.98	.03
Effect per 1 SD	1.06	0.91-1.23	.45	1.04	0.95-1.13	.40	0.79	0.66-0.94	.007	0.83	0.75-0.93	.001

AHEI = the Alternative Healthy Eating Index; CI = confidence interval; CVD = cardiovascular disease; OR = odds ratio. Model 1: Adjusted for age, sex and total energy intake. Model 2: Model 1 + additionally adjusted for smoking habits and physical activity. Results are from logistic regression models estimating the association of adherence to AHEI at baseline with 4 aging outcomes at follow-up: ideal health (n = 213), nonfatal cardiovascular disease (n = 680), cardiovascular death (n = 149), and noncardiovascular death (n = 392). From the distribution of these factor scores, participants were categorized into tertiles for each pattern: the Tertile 1 group included participants with a factor score below the 33^rd^ percentile, the Tertile 2 group included participants with a factor score in the 33^th^-66^th^ percentile range and the Tertile 3 group included those with a factor score above the 66^th^ percentile.

## Clinical Significance

• Data from British adults suggest that avoidance of a diet comprising fried and sweet food, processed and red meat, refined grains, and high-fat dairy products is associated with a higher chance of achieving older ages disease-free and highly functional.
• Adherence to dietary recommendations provided by the Alternative Healthy Eating Index is associated with reduced risk of premature death.
